# Development of the RF-MEAM Interatomic Potential for the Fe-C System to Study the Temperature-Dependent Elastic Properties

**DOI:** 10.3390/ma16103779

**Published:** 2023-05-17

**Authors:** Sandesh Risal, Navdeep Singh, Andrew Ian Duff, Yan Yao, Li Sun, Samprash Risal, Weihang Zhu

**Affiliations:** 1 Department of Mechanical Engineering, University of Houston, Houston, TX 77204, USA; srisal@cougarnet.uh.edu (S.R.); lsun4@uh.edu (L.S.); 2 Department of Mechanical Engineering, School of Engineering and Computer Science, University of the Pacific, Stockton, CA 95211, USA; 3 Scientific Computing Department, STFC Daresbury Laboratory, Warrington WA4 4AD, UK; andrew.duff@stfc.ac.uk; 4 Materials Science and Engineering Program, University of Houston, Houston, TX 77204, USA; yyao4@central.uh.edu (Y.Y.); srisal2@cougarnet.uh.edu (S.R.); 5 Department of Electrical and Computer Engineering, University of Houston, Houston, TX 77204, USA; 6 Department of Engineering Technology, University of Houston, Houston, TX 77204, USA

**Keywords:** RF-MEAM, steel, iron-carbon, molecular dynamics, density functional theory, MEAMfit, inter-atomic potential

## Abstract

One of the major impediments to the computational investigation and design of complex alloys such as steel is the lack of effective and versatile interatomic potentials to perform large-scale calculations. In this study, we developed an RF-MEAM potential for the iron-carbon (Fe-C) system to predict the elastic properties at elevated temperatures. Several potentials were produced by fitting potential parameters to the various datasets containing forces, energies, and stress tensor data generated using density functional theory (DFT) calculations. The potentials were then evaluated using a two-step filter process. In the first step, the optimized RSME error function of the potential fitting code, MEAMfit, was used as the selection criterion. In the second step, molecular dynamics (MD) calculations were employed to calculate ground-state elastic properties of structures present in the training set of the data fitting process. The calculated single crystal and poly-crystalline elastic constants for various Fe-C structures were compared with the DFT and experimental results. The resulting best potential accurately predicted the ground state elastic properties of B1, cementite, and orthorhombic-Fe${}_{7}$C${}_{3}$ (O-Fe${}_{7}$C${}_{3}$), and also calculated the phonon spectra in good agreement with the DFT-calculated ones for cementite and O-Fe${}_{7}$C${}_{3}$. Furthermore, the potential was used to successfully predict the elastic properties of interstitial Fe-C alloys (FeC-0.2% and FeC-0.4%) and O-Fe${}_{7}$C${}_{3}$ at elevated temperatures. The results were in good agreement with the published literature. The successful prediction of elevated temperature properties of structures not included in data fitting validated the potential’s ability to model elevated-temperature elastic properties.

## 1. Introduction

Steel is a prevalent material in our everyday life, with a broad range of applications due to its widespread availability and desirable mechanical properties. High-temperature mechanical properties of steel are of immense interest to the community dedicated to understanding and designing steel [1,2,3,4,5,6,7,8,9]. The investigation of high-temperature properties through physical experimentation can be a challenging and resource-intensive process, making it a costly pursuit for researchers. The use of computational tools has significantly impacted the field of high-temperature research by providing a cost-effective and time-efficient alternative for exploring material properties; while there are limitations to the accuracy and scope of these calculations, recent advances in computational ability have made it possible for researchers to obtain reliable results that are on par with physical experiments. As such, computational methods have become a valuable tool for investigating high-temperature phenomena and complementing traditional experimental approaches.

Density Functional Theory (DFT) is considered one of the most accurate computational tools for quantum chemistry calculations and has been employed to predict a wide range of material properties of the many-body system [10,11,12,13]. However, due to the high computational requirement, their use is limited to smaller systems, typically containing a few hundred atoms. To overcome this limitation of DFT calculations, molecular dynamics (MD) calculations using interatomic potentials are used to model bigger systems. The interatomic interactions in such calculations are modeled using empirical or semi-empirical potentials. The performance and accuracy of results depending entirely on the quality of these potentials [14,15]. Hence, the focus of many researchers has been to increase the quality of the interatomic potentials.

Over the years, various interatomic potential formulations have been proposed. The Embedded Atom Method (EAM) and the Modified Embedded Atom Method (MEAM) are widely used formalisms for metals and alloys [16,17,18,19,20]. Daw and Baskes developed EAM [21] in 1983 and demonstrated the potential’s ability to study fracture caused by hydrogen embrittlement in transition metals. Since then, it has been extensively used to investigate a variety of phenomena, such as point defects, melting, alloying, grain boundary structure and energy, fracture, segregation, surface structure, and epitaxial growth [22,23,24]. THe EAM formulation was modified to include angular dependencies of electron density to develop the MEAM potential. The addition of the angular dependence term in MEAM has the advantage of accurately defining the interactions in metals and their alloys. It was first used to derive semi-empirical potentials for silicon, germanium, and their alloys [25]. MEAM has been further improved to a more accurate formalism called Second Nearest Neighbor (2NN) MEAM by Lee and Baskes [26]. This semi-empirical model accurately describes the physical properties of a wide range of elements and alloys. In 2NN MEAM formalism, the interatomic potential is calculated from total energy and embedding energy function (refer to Equation (4)). Then, the total energy is obtained analytically from the zero-temperature Rose universal equation of state [27], which was further modified by Li et al. [28], as shown in Equations (1)–(3). Hence, the interatomic potential is tied to an equilibrium reference structure.

(1) $$E_{i}^{u}\left(R\right)=-E_{i}^{0}\left(1+a^{*}+a_{3}\frac{a^{*}}{\left(R/R_{i}^{0}\right)}\right)e^{-a^{*}}$$

(2) $$a^{*}=\alpha_{i}\left(\frac{R}{R_{i}^{0}}-1\right)$$

(3) $$\alpha_{i}=\sqrt{\frac{9B_{i}\Omega_{i}}{E_{i}^{0}}}$$

where *R* is the nearest neighbor distance, $R_{i}^{0}$ is the equilibrium nearest neighbor distance, $E_{i}^{0}$ is the cohesive energy, $B_{i}$ is the bulk modulus, and $\Omega_{i}$ is the equilibrium volume of the reference structure.

Another version of MEAM is the reference-free MEAM (RF-MEAM) formalism [29]. In contrast to conventional MEAM, the potential in RF-MEAM formalism is no longer bound to the Rose equation, and the pair interactions are defined using explicit functions with adjustable parameters that can be optimized during the fitting process. The energies of any structure can hence be reproduced by fitting a handful of parameters without defining a reference structure. Hence, this formalism is called reference-free MEAM (RF-MEAM).

For the Fe-C system, various semi-empirical interatomic potentials have been developed to study different physical properties. Johnson et al. [30] and Rosato [31] were among the first to develop a potential for the Fe-C system. These studies were focused on the effect of intrusion of carbon atoms in the Fe-host structure. Since the potential did not account for carbon–carbon interactions, they were limited only to the study of material properties related to solute–solvent interactions. Later, Ruda et al. [32] developed an EAM potential for Fe-C that could efficiently predict the heat of solution of C in Fe as well as account for C-C interactions. Henriksson et al. [33] also developed an analytical bond order interatomic potential for Fe-C capable of predicting simple point defects in carbides in reasonable agreement with the available experimental and DFT data. Later, Lee [34] developed an interatomic potential for Fe-C based on the previously developed 2NN MEAM potentials for Fe and C. Using this potential, various physical properties, such as the dilute heat of solution of carbon, the vacancy–carbon binding energy, and its configuration, the location of interstitial carbon atoms and the migration energy of carbon atoms in bcc and fcc Fe were calculated with good accuracy. In 2014, Lalitha et al. [35] developed a potential for Fe-C based on the MEAM formalism and successfully predicted the melting temperature of cementite.

Although many potentials [30,31,32,34,35,36,37,38] have been developed for iron-carbon alloys, there are no such potentials that are dedicated to the high-temperature elastic properties. In addition, iron-carbon alloys have not been studied using RF-MEAM formalism. Thus, we aim to produce an inclusive potential for the Fe-C system within the framework of the RF-MEAM formalism that can simulate and reproduce high-temperature elastic properties of different phases with good accuracy.

## 2. Materials and Methods

In the RF-MEAM formalism, the total energy of the N-atom system has a form similar to EAM potential and is expressed as [39]:

(4) $$E=\sum_{i=1}^{N}E_{i}^{emb}\left(\rho_{i}\right)+\frac{1}{2}\sum_{i\neq j}^{N}\phi_{ij}\left(r_{ij}\right)$$

where $E_{i}^{emb}\left(rho_{i}\right)$ is the embedding function, $\rho_{i}$ is the fictitious electron density at site *i*, and $\phi_{ij}\left(r_{ij}\right)$ is the pair potential between atoms *i* and *j* separated by a distance $r_{ij}$. The embedding function is described using Equations (5)–(7) [40]:

(5) $$E_{i}^{emb}\left(\rho_{i}\right)=a_{i}\rho_{i}^{\frac{1}{2}}+b_{i}\rho_{i}^{2}+c_{i}\rho_{i}^{3}$$

(6) $$\rho_{i}=\frac{2\rho_{i}\left(0\right)}{1+e^{-\tau_{i}}}$$

(7) $$\tau_{i}=\sum_{l=1}^{3}t_{i}^{\left(l\right)}{\left(\frac{\rho_{i}^{\left(l\right)}}{\rho_{i}^{\left(0\right)}}\right)}^{2}$$

where $a_{i}$, $b_{i}$, and $c_{i}$ are (species-dependent) parameters that need to be optimized and $\rho_{i}^{\left(0\right)}$ and $\rho_{i}^{\left(l\right)}$ are contributions to background density $\rho_{i}$ with and without angular contribution, respectively, which are again modeled using the following Equations [26]:

(8) $$\rho_{i}^{\left(0\right)}=\sum_{j\neq i}^{N}f_{i}^{\left(0\right)}\left(r_{ij}\right)$$

(9) $$\rho_{i}^{l}=\sum_{j,k(\neq i)}^{N}f_{i}^{\left(l\right)}\left(r_{ij}\right)f_{k}^{l}\left(r_{ik}\right)P^{\left(l\right)}\left(cos\theta_{jik}\right)$$

where $P^{\left(l\right)}\left(cos\theta_{jik}\right)$ is the Legendre polynomial which introduces the effect of bond angles into the MEAM formalism. The terms $f_{j}^{\left(l\right)}$ and $f_{k}^{\left(l\right)}$ are the partial background density contributions, which are described by Equation (10) [26]:

(10) $$f_{j}^{l}\left(r\right)=\sum_{n=1}^{N_{1}}a_{j}^{\left(n,l\right)}{\left(r_{j}^{\left(n,l\right)}-r\right)}^{3}\cdot \theta \left(r_{j}^{\left(n,l\right)}-r\right)$$

Similarly, the pair potential is expressed as shown in Equation (11) [41]:

(11) $$\phi_{i,j}\left(r_{ij}\right)=\sum_{n=1}^{N2}b_{i,j}^{\left(n\right)}{\left(s_{i,j}^{\left(n\right)}-r\right)}^{3}\cdot \theta \left(s_{i,j}^{\left(n\right)}-r\right)$$

where *n* is the total number of terms to be included in the pair potential and electron density, $a_{j}^{\left(n,l\right)}$, $r_{j}^{\left(n,l\right)}$, $b_{i,j}^{\left(n\right)}$, and $s_{i,j}^{\left(n\right)}$ are the parameters that need to be optimized, $\theta \left(r_{j}^{\left(n,l\right)}-r\right)$, and $\theta \left(s_{i,j}^{\left(n\right)}-r\right)$ are the step functions in the form $\theta (r^{{}^{\prime }}-r)$ with cutoff $r^{{}^{\prime }}>r$. For a two-atom system, considering three pairwise terms for each of the electron densities and pair-potential (i.e., $N_{1}=N_{2}=3$ in Equations (10) and (11)), there will be 72 parameters in the potential file which will be fitted on the energies, forces, and stress tensors data.

**Figure 1:**
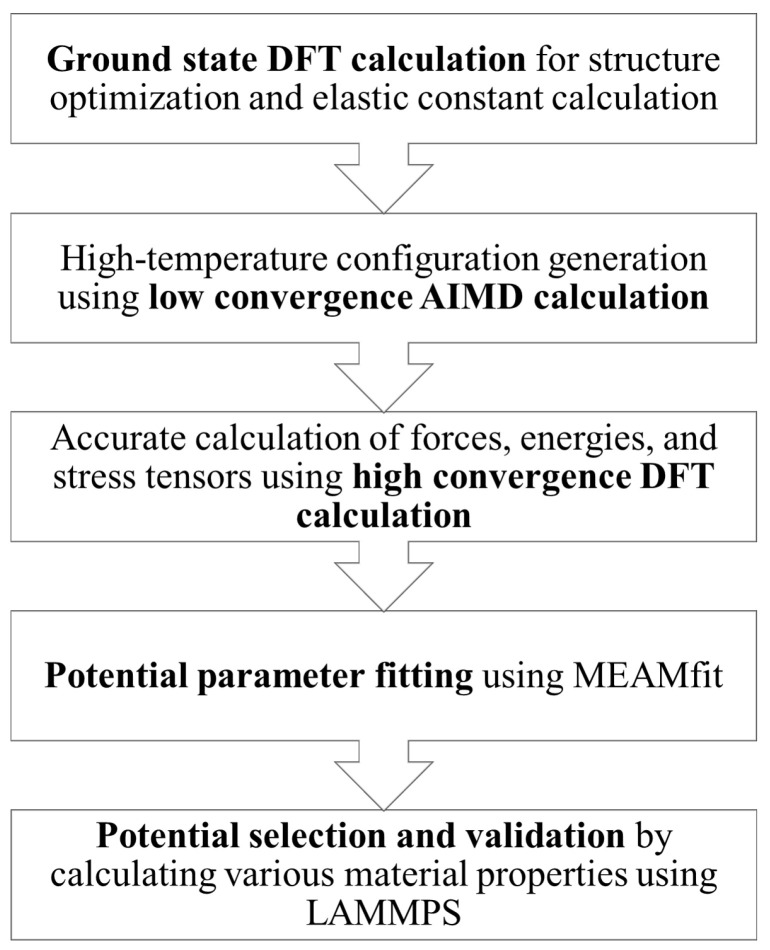
Flowchart of the methodology used for the potential development.

Four common hypothetical structures of Fe-C (B1, B2, B3, and L12) and one experimentally observed structure (cementite) were initially considered for the calculation. The methodology used in this work is shown in Figure 1 and is discussed further below.

### 2.1. Ground State DFT Calculation

All DFT calculations were performed employing the Vienna Ab-initio Simulation Package (VASP) [42] using projector augmented wave (PAW) [43] pseudopotentials. The exchange-correlation functional was calculated using the Perdew–Burke–Ernzerhof Generalized-Gradient Approximation (GGA-PBE) [44] due to its efficiency and accuracy in describing the properties of transition metals and their alloys, particularly for elastic properties [45,46,47,48]. The ground state energy was calculated for all five structures by performing zero Kelvin structure optimization, for which the number of atoms, supercell structures, and *k*-points used is given in Table 1. Similarly, the structures to be used for AIMD calculations were also optimized, for which the number of atoms, supercells, and *k*-points is shown in Table 2. As seen in the tables, larger supercells were used for accurate ground-state energy calculations. However, smaller supercells were used for the optimization prior to AIMD because performing DFT and AIMD calculations using the larger supercells would be computationally expensive. For optimization, a pre-defined calculation sequence was performed.

In addition to structure optimization, elastic constants calculation was also performed on the structures in Table 1. The elastic tensors were obtained by applying six finite distortions to the lattice and using the resulting stress–strain relationship to calculate the elastic constants. The calculated elastic constants were later used for the comparison with the MD calculated result. The energies, forces, and stress tensors from the elastic constant calculations were also used as the input in the data fitting process.

Phonon calculations using DFT were performed implementing the finite difference method using the PHONOPY code [49]. The code calculates the Hellmann–Feynman forces induced by single atom displacement calculated from the supercells through the VASP code. For cementite, a 3 × 2 × 2 supercell with 192 atoms was used, whereas for O-Fe${}_{7}$C${}_{3}$, a 2 × 2 × 1 supercell with 240 atoms was used. The cutoff energy for the phonon dispersions calculation was 520 eV, and the *k*-points of brillouin zone sampling grid of 4 × 3 × 3 and 3 × 3 × 3 was applied for cementite and O-Fe${}_{7}$C${}_{3}$, respectively.

### 2.2. Low-Convergence AIMD Calculation

The optimized structures, as discussed in Section 2.1, were used to perform finite temperature AIMD calculations to obtain various high-temperature configurations. It is important to note that the purpose of these calculations is not to accurately calculate forces, energies, and stress tensors, but rather to perform phase sampling. This was accomplished by performing AIMD calculations using low-convergence criteria, such as the “Fast” Algorithm, “Normal” precision, and a single *k*-point, i.e., 1 × 1 × 1. Initially, MD runs were performed using an NPT ensemble for 500 steps with a time step of 1.5 fs at a temperature of 300 K and a pressure of 1 Bar. These equilibration steps ensure that the simulations start from a consistent and well-defined initial condition, allowing for accurate and reliable results. After system equilibration, data were collected by performing an additional 1000 steps at NPT, referred to as the production run. Next, thirty configurations were randomly extracted from these production steps for the high-convergence calculations. The same method was repeated for ionic temperatures of 500 K and 1000 K.

### 2.3. High-Convergence DFT Calculation

From the AIMD calculation results, thirty random configurations were taken for each of the five structures (see Table 1) at temperatures of 300 K, 500 K, and 1000 K, resulting in a total of 450 random configurations. For each of these configurations, high-convergence criteria were used for precise calculation of forces, energies, and stress tensors. High-convergence criteria are cut-off energy of 520 eV, EDIFF (energy difference criteria for exiting the electronic convergence loop) equal to $10^{-6}$ eV, and denser *k*-points, which are given in Table 2. This technique of using low parameters to efficiently sample the phase space and then using higher parameters to obtain accurate observables is similar to the ‘upsampled thermodynamic integration using Langevin dynamics’ (UP-TILD) method [50] described by Srinivasan et al. in [51].

### 2.4. Potential Parameter Fitting

Structure optimization, AIMD calculation, and high-convergence calculation, collectively, can be referred to as “data generation”. The data obtained from the high-convergence calculations, as well as the data from elastic constant calculations, were an input for the MEAMfit code developed by Andrew Ian Duff [52]. The “vasprun.xml” files generated in each DFT calculation were used as input files for data fitting. MEAMfit code extracts energies, forces, and stress tensors data from “vasprun.xml” files and outputs EAM or RF-MEAM potentials parameter files.

Multiple datasets were formed from the data of five structures and were used independently in the data fitting process. Three summation terms were selected for the partial electron densities and the pair potential during the fitting process. A weight ratio of 1:0.1:0.001 was used for the energies, atomic forces, and stress tensors. In the case of the comprehensive dataset (i.e., a dataset containing all five structures’ data), 78 parameters of RF-MEAM were fitted to 40,805 data points. For each dataset, ten potentials were generated and further optimized using a genetic algorithm and conjugate-gradient minimization scheme. The optimization function was calculated for the newly developed potentials during the optimization process, and if the optimization function is smaller than any of the previously calculated top-ten potentials, it replaced that potential in the list. This process was repeated until the convergence criteria are met. Finally, the potential with the smallest optimization function, R, was chosen as the optimal potential from each dataset.

### 2.5. Potential Selection and Validation

The optimized potentials were employed to perform MD calculations on LAMMPS (an adapted version of LAMMPS for RF-MEAM by Prasanth Srinivasan [51]) to predict the ground-state mechanical properties of the alloys. Initially, the generated potentials were used to calculate the equilibrium volume, and bulk modulus using the Birch–Murnaghan equation of state, compliance tensors, and elastic constants, of various Fe-C structures. The calculated properties were then compared with the DFT and experimental results, published in the literature. In other words, the potential that could precisely predict the mechanical properties of known alloys (ones used in the fitting process) of Fe-C was termed as the best potential.

The final potential from the abovementioned process was used to perform phonon calculation to reproduce the phonon dispersion curves for cementite and orthorhombic-Fe${}_{7}$C${}_{3}$ (hereafter referred to as O-Fe${}_{7}$C${}_{3}$) structures. The potential-level phonon calculations were performed using phonoLAMMPS [53], which calculate the harmonic interatomic force constants using phonopy and LAMMPS. Additionally, the potential was tested to predict the elastic properties of the structures that were not included in the data fitting process. Specifically, Young’s modulus, bulk modulus, and rigidity modulus of elasticity at elevated temperatures were calculated for Fe-C alloys containing 0.1% and 0.2% of carbon by weight (hereafter referred to as FeC-0.1%, and FeC-0.2%, respectively), and O-Fe${}_{7}$C${}_{3}$ alloys, after which they were compared to the experimental results that have been previously reported in the literature.

## 3. Results and Discussion

### 3.1. Parameters for AIMD Calculations

The ab initio MD calculations mentioned in the methods section were performed using an NPT ensemble. An NPT ensemble in VASP is attained by combining the Parrinello–Rahman [54] method with a Langevin thermostat [55]. The NPT ensemble calculations require the specification of the following parameters: LANGEVIN_GAMMA, which sets the friction coefficient for the atomic degrees-of-freedom for Langevin thermostat; LANGEVIN_GAMMA_L, which sets the friction coefficient for lattice degrees-of-freedom for Langevin thermostat using the Parrinello–Rahman method; and PMASS, which sets a fictitious mass to the lattice degrees-of-freedom in case of the Parrinello–Rahman method. Since the above-mentioned parameters are system-dependent, they need to be defined for each system. The best estimate of these parameters has been determined by the trial-and-error method, as discussed further.

**Figure 2:**
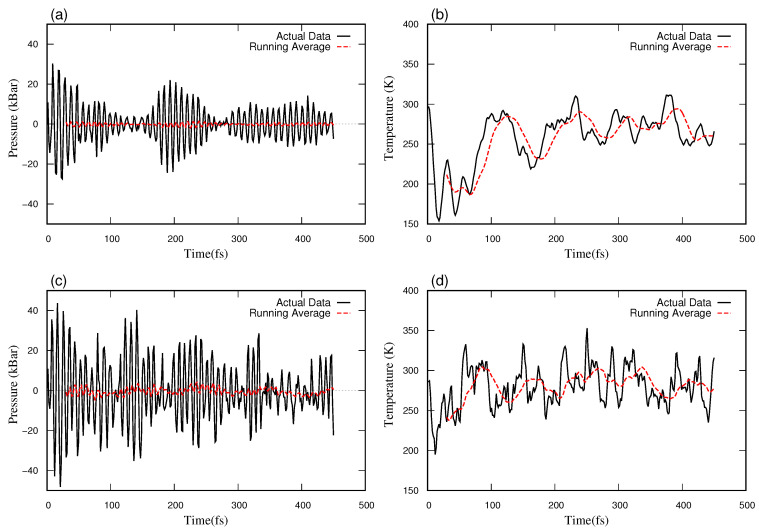
Fluctuations in regulated parameters over time in the NPT ensemble using LANGEVIN_GAMMA and LANGEVIN_GAMMA_L equal to 0.5 and PMASS equals 1: (**a**) Pressure vs. time, (**b**) Temperature vs. time, where LANGEVIN_GAMMA and LANGEVIN_GAMMA_L equal to 50 and PMASS equals to 1: (**c**) Pressure vs. time, (**d**) Temperature vs. time. The black (solid) line is the actual data at a certain time, whereas the red (dotted) line is the running average over every 20 datapoints.

We started by fixing the value of LANGEVIN_GAMMA and LANGEVIN_GAMMA_L to 0.5, and systematically changing the value of PMASS to 1, 3, 5, 10, 100, and 1000. Temperature and pressure data at each step were extracted from the OUTCAR file and root mean squared error (RMSE) on both temperature and pressure was calculated for each case. The RMSE quantifies the average deviation of the calculated instantaneous temperatures and pressures from the target values of 300 K and 0 bar, respectively. The RMSEs of temperature and pressure were independently ranked for each case, and the average of these two rankings was subsequently calculated for each case. The case with PMASS = 1 was found to have the least averaged rank, for which the temperature and pressure fluctuations are shown in Figure 2a,b. Again, fixing PMASS to 1, LANGEVIN_GAMMA and LANGEVIN_GAMMA_L were varied (1, 3, 5, 10, 20, 50, and 100). This process gave an optimized set of these parameters. The least averaged rank was obtained at PMASS = 1, and LANGEVIN_GAMMA and LANGEVIN_GAMMA_L were both equal to 50. The pressure and temperature fluctuations in calculation using these optimized parameters are shown in Figure 2c,d.

### 3.2. Ground State Elastic Calculation

**Figure 3:**
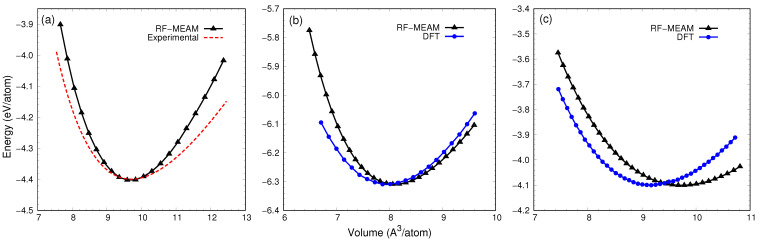
Comparison of energy vs. volume curves for (**a**) cementite, (**b**) B1, and (**c**) L12 with experimental and DFT results. Both DFT and experimental results used for the comparison are from Lalitha et al. [35]. These curves are shifted vertically to match the RF-MEAM calculated equilibrium energy.

Energy versus volume data was obtained for B1, B2, B3, cementite, and O-Fe${}_{7}$C${}_{3}$ structures using the potentials developed from different datasets. By fitting the energy and volume data to the Birch–Murnaghan equation of state, the bulk modulus of elasticity and its pressure derivative were obtained for each potential. These results, along with the elastic tensors presented later in this section, were compared to experimental and DFT results in order to assess the accuracy and reliability of the potentials, which allowed us to identify the best potential. Table 3 details the results produced by our best RF-MEAM potential in comparison to the experimental and DFT results from the literature. From the table, it is apparent that the results from our best potential are in very good agreement with the literature having a deviation of 2.7%, 11.9%, 11.6%, 7.7%, and 9.5% in bulk modulus for B1, B2, B3, cementite, and Fe${}_{7}$C${}_{3}$, respectively. Figure 3a illustrates the change in cohesive energy as a function of volume for cementite. The experimental curve was obtained by substituting experimental values of V${}_{0}$, B${}_{0}$, and B${}_{0}$’ in Murnaghan’s equation of state, as mentioned in [35]. As seen in the figure, the RF-MEAM potential predicts the equilibrium volume in good agreement with the experimental curve (≈1.2% deviation). Similarly, Figure 3b compares the cohesive energy versus volume curve for B1 calculated using the RF-MEAM potential with the DFT curve reported by [35], showing that the equilibrium volume calculated using the RF-MEAM potential is approximately 2.4% more than in the DFT curve. Figure 3c also compares the cohesive energy versus volume curve for the L12 structure calculated using the RF-MEAM potential with its DFT-calculated counterpart, showing that the equilibrium volume calculated using the RF-MEAM is approximately 5.8% more than in the DFT curve. In all three cases, for ease of comparison, the experimental or DFT curves are shifted vertically to match the minimum energy obtained using the RF-MEAM potential.

Stress–strain relationships were evaluated to calculate the compliance tensor by distorting the lattice in six different directions independently. These compliance tensors satisfied the generalized stability criteria for cubic and orthorhombic crystals [60]. Polycrystalline elastic constants were calculated from these single-crystal elastic constants using the Reuss, Voigt, and Hill Equation [61,62,63]. More details on polycrystalline modulus calculations are provided in the supplementary information. Table 4 shows the compliance tensor and polycrystalline elastic properties (bulk modulus, Young’s modulus, and rigidity modulus) in comparison with the results from different studies.

For cementite, the MD-calculated compliance tensors are comparable to the DFT-calculated counterparts and have a minimal deviation from the published results. The bulk modulus (B) calculated by the RF-MEAM potential is within 28.4% of the DFT result from the literature. However, the rigidity modulus (G) and Young’s modulus (Y) are within 2.1% and 1.2%, respectively. For B1, the compliance tensors as well as the polycrystalline elastic moduli are in very good agreement with the literature results. The deviation of the calculated polycrystalline elastic moduli w.r.t. the literature results is less than 5%. Additionally, in the case of O-Fe${}_{7}$C${}_{3}$, the potential was able to faithfully reproduce the single crystal elastic constants, even though this structure has not been used in the fitting process. The polycrystalline elastic constants for O-Fe${}_{7}$C${}_{3}$, namely B, G, and Y, also exhibit a similar trend and are in good agreement with the published results.

**Figure 4:**
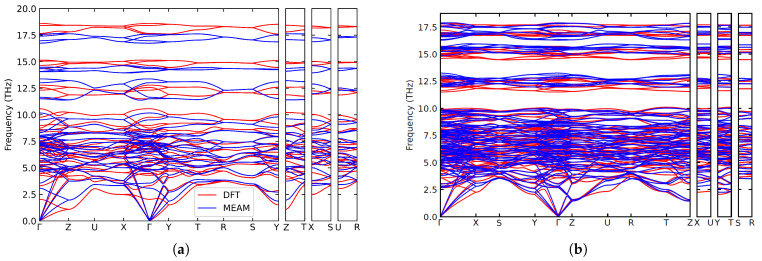
Comparison of phonon dispersion curves calculated with the optimized potential (blue) with the ones calculated with DFT (red) for (**a**) cementite and (**b**) O-Fe${}_{7}$C${}_{3}$ structures. For a color version of this figure, please refer to the online version of this article.

For further validation, the phonon spectra were computed at the DFT level and using the optimized potential. Spectra were computed for cementite, O-Fe${}_{7}$C${}_{3}$, and B1 phases. The B1 phase was found to be dynamically unstable, with imaginary frequencies present in the spectra (not shown here). Cementite and O-Fe${}_{7}$C${}_{3}$ were both dynamically stable, and as shown in Figure 4, the spectra computed using the potential are in good agreement with the DFT results for both phases. This includes the slopes at the Gamma point, which are related to the elastic constants, explicitly included in the training set. Other features of the spectra were not directly included in the training process, and the good performance of the potential here validates the use of molecular dynamics simulations in the DFT training set for effectively capturing the vibrational properties of the materials under study.

### 3.3. Finite-Temperature Elasticity Calculation

Elastic properties of interstitial alloy Fe-C with bcc structure at various temperatures were reproduced using the developed potential. Two alloys of Fe-C, with 0.2% and 0.4% carbon concentration by weight, were considered for this study. These alloys were created by randomly placing carbon atoms in octahedral interstitial positions of the bcc-Fe structure. The number of carbon atoms to be placed were calculated by converting the weight percentage to atomic percentage. For Fe-C-0.2% and 0.4% alloys, the conversion would be ≃1, and ≃2 atomic percentages, respectively. The individual elastic constants Cij were calculated with the developed potential and usinzg the Reuss, Voigt, and Hill approximation [65], while polycrystalline elastic constants were also calculated, as shown in Table 5. Furthermore, the aforementioned mechanical stability criteria for the cubic structure were satisfied by the individual elastic constants.

**Figure 5:**
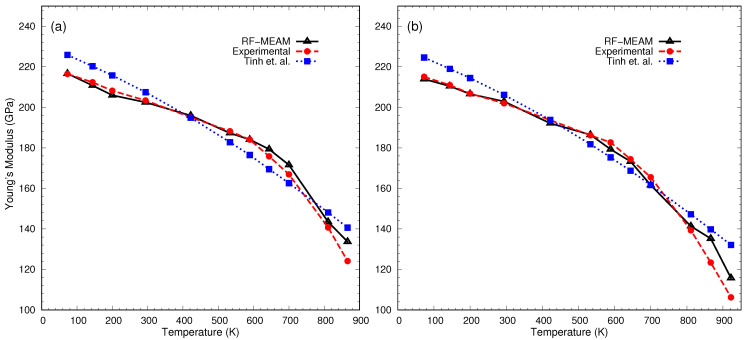
Young’s moduli of interstitial Fe-C alloys: (**a**) FeC-0.2% and (**b**) FeC-0.4% at various temperatures. For comparison, experimental data [66] and SMM-calculated data by Tinh et al. [67] are also included.

The calculated Young’s moduli of elasticity for FeC-0.2% and FeC-0.4% were compared with the experimental data reported by [66] and the SMM (Statistical Moment Method) calculated data reported by Tinh et al. [67]. The comparison is depicted using the graph in Figure 5. As seen in Figure 5a, for FeC-0.2%, the calculated Young’s moduli at different temperatures precisely follow the experimental results with a maximum deviation of 7.7% at 866 K. In Figure 5b, the Young’s moduli for FeC-0.4% have been plotted against the respective temperatures. The Young’s modulus decreased with increasing temperatures and closely follows the experimental curve. The maximum deviation in the case of FeC-0.4% alloy was 13.3% at 866 K. It is also worth mentioning that for both alloys, our potential was able to reproduce the Young’s modulus for a wide range of temperatures more accurately than previously reported results.

**Figure 6:**
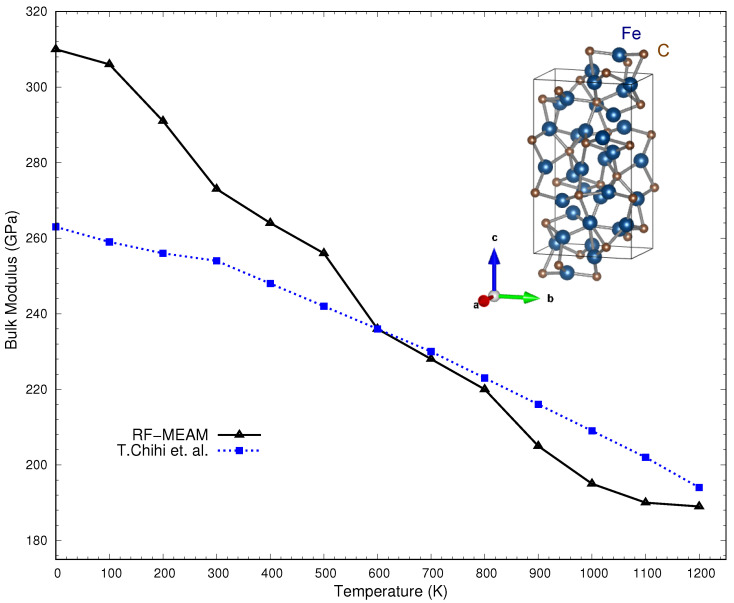
Bulk moduli of O-Fe${}_{7}$C${}_{3}$ at various temperatures (up to 1200 K) in comparison with the DFT results of T. Chihi et al. [57].

Figure 6 shows the bulk modulus for the O-Fe${}_{7}$C${}_{3}$ alloy as a function of temperature, with the DFT result by T. Chihi et al. [57] included alongside the comparison. The results were compared with DFT data rather than experimental due to the unavailability of robust experimental data for the structure. As shown in the figure, the calculated bulk modulus deviates noticeably from T. Chihi et al.’s result at low temperatures. However, at higher temperatures, the results are in excellent agreement, from room temperature up to 1200 K. The slope of the calculated curve seems to be overestimated initially. However, above room temperature, the slope aligns better with the DFT-calculated results. A point of note here is that the produced potential predicted the elevated temperature bulk modulus as precisely as DFT-calculated results.

## 4. Conclusions

In summary, we developed a potential for the Fe-C alloy system within the RF-MEAM formalism by fitting the parameters of the potential against the DFT-calculated energies, forces, and stress tensor data. Various structures were considered for creating eclectic datasets. The potential was developed to reproduce the elastic properties of Fe-C alloy system, especially at elevated temperatures. To test the potential, elastic properties of B1, cementite, and O-Fe${}_{7}$C${}_{3}$ were calculated and compared against our DFT calculation results, as well as those reported in the literature (experimental, DFT, and MD results). The comparison revealed good agreement, indicating the effectiveness of the potential. Furthermore, we also compared the phonon spectra calculated using the potential with the DFT-calculated phonon dispersion curves; the potential-calculated curve closely followed the DFT-calculated curve, demonstrating good agreement between the two methods. In addition to testing the accuracy of the potential to predict ground state properties, we reproduced the elastic properties of Fe-C alloys at higher temperatures. Specifically, we examined FeC-0.2%, FeC-0.4%, and O-Fe${}_{7}$C${}_{3}$. The results showed that the RF-MEAM potential was able to accurately predict the elastic properties of these materials up to 1200 K, with some predictions even surpassing previously reported literature’s accuracy.

We believe that this work will lay the foundation for further investigation into the use of the RF-MEAM potential for predicting the elastic properties of steel at a range of temperatures and compositions. In future work, we will be focusing on adding more major constituent elements of steel, such as Mn, Si, Cr, etc., to form a more broadly applicable Rf-MEAM potential. Additionally, it would be useful to understand the underlying factors that contribute to the potential’s accuracy, in order to further improve its predictive power. Overall, our research has shown that the RF-MEAM potential is promising for predicting elastic properties in steel alloys, and has the potential to be a valuable asset for materials’ design and modeling.

## Figures and Tables

**Table 1:** Description of various structures used for ground state calculations. The unit cells of each structure are detailed in supplementary information (Figure S1).

Fe-C Structure	*k*-Points (Gamma)	No. of Atoms	Supercell
B1	7 × 15 × 15	128	4 × 2 × 2
B2	9 × 12 × 16	120	5 × 4 × 3
B3	7 × 15 × 15	128	4 × 2 × 2
Cementite	23 × 10 × 7	64	1 × 2 × 2
L12	10 × 15 × 15	48	3 × 2 × 2

**Table 2:** Description of various structures used for AIMD calculations.

Fe-C Structure	*k*-Points (Gamma)	No. of Atoms	Supercell
B1	15 × 15 × 15	64	2 × 2 × 2
B2	16 × 16 × 16	54	3 × 3 × 3
B3	15 × 15 × 15	64	2 × 2 × 2
Cementite	23 × 10 × 7	64	1 × 2 × 2
L12	10 × 15 × 15	48	3 × 2 × 2

**Table 3:** Comparison of our results with the published DFT and experimental data for equilibrium volume, bulk modulus, and pressure derivative calculated by the Birch–Murnaghan equation of state. Experimental data are included in the brackets.

Structure	V${}_{0}$ (Å${}^{3}$)		B${}_{0}$ (GPa)		B${}_{0}$’	
	RF-MEAM	Literature	RF-MEAM	Literature	RF-MEAM	Literature
B1 (FeC)	65	$64{\;}^{a,b}$	338	$329{\;}^{a}$	5.28	$4.40{\;}^{a}$
B2 (FeC)	16	$15{\;}^{a}$	302	$343{\;}^{a}$	4.12	$4.40{\;}^{a}$
B3 (FeC)	78	$77{\;}^{a}$	222	$251{\;}^{a}$	4.50	$4.20{\;}^{a}$
Cementite (Fe3C)	155	$154{\;}^{a}$	252	$234{\;}^{a}$	4.05	$4.00{\;}^{a}$
O-Fe${}_{7}$C${}_{3}$	94	$89{\;}^{b}$, $91{\;}^{c}$ ($93{\;}^{d}$)	288	$263{\;}^{b}$, $262{\;}^{a}$	3.10	$5.04{\;}^{b}$, $3.70{\;}^{a}$

${}^{a}$ Henriksson et al. [56]; ${}^{b}$ Chihi et al. [57]; ${}^{c}$ Fang et al. [58]; ${}^{d}$ Villars [59] as cited by Henriksson et al. [56].

**Table 4:** Single-crystal and polycrystalline elastic constants for different alloys calculated using the produced RF-MEAM potential. Elastic constants of other alloys (B2, B3, and L12) are included in the Supplementary Information’s Table S1.

Elastic Constants (GPa)	Fe3C-Cementite			FeC-B1			O-Fe${}_{7}$C${}_{3}$		
	This Study		Literature	This Study		Literature	This Study		Literature
	RF-MEAM	DFT	DFT/MEAM	RF-MEAM	DFT	DFT/MEAM	RF-MEAM	DFT	DFT/MEAM
C11	399	296	$388{\;}^{a}$	585	584	$566{\;}^{b}$	399	344	$394{\;}^{c}$
C22	459	392	$345{\;}^{a}$	-	-	-	469	428	445c
C33	364	330	$322{\;}^{a}$	-	-	-	444	423	$452{\;}^{c}$
C12	236	137	$156{\;}^{a}$	214	205	$213{\;}^{b}$	238	166	$185{\;}^{c}$
C13	210	181	$164{\;}^{a}$	-	-	-	245	170	$179{\;}^{c}$
C23	204	155	$162{\;}^{a}$	-	-	-	255	182	$170{\;}^{c}$
C44	116	131	$134{\;}^{a}$	125	82	$145{\;}^{b}$	106	95	$126{\;}^{c}$
C55	40	19	$15{\;}^{a}$	-	-	-	98	89	$112{\;}^{c}$
C66	119	135	$134{\;}^{a}$	-	-	-	81	66	$78{\;}^{c}$
						-			
B	280	218	$224{\;}^{a}$	338	331	$331{\;}^{b}$	310	247	$262{\;}^{c}$
G	93	93	$95{\;}^{a}$	149	125	$158{\;}^{b}$	95	95	$114{\;}^{c}$
Y	252	245	$249{\;}^{a}$	390	334	$408{\;}^{b}$	259	253	$298{\;}^{c}$

${}^{a}$ DFT result from Jiang et al. [64]; ${}^{b}$ Result from MEAM potential by Laalitha et al. [35]; ${}^{c}$ DFT result from Chihi et al. [57].

**Table 5:** Various elastic moduli of FeC-0.2% and FeC-0.4% alloys at different temperatures.

T(K)	FeC-0.2%			FeC-0.4%		
	B (GPa)	G (GPa)	E (GPa)	B (GPa)	G (GPa)	E (GPa)
73	215	81	217	214	80	214
144	211	79	211	210	79	210
200	206	77	206	204	78	207
294	202	76	203	202	76	203
422	195	74	196	194	72	192
533	186	70	187	185	70	186
589	186	69	184	183	67	179
644	179	67	179	180	65	173
700	173	64	172	174	60	162
811	167	53	144	169	52	141
866	165	49	134	162	50	135
922	-	-	-	154	42	116

## Data Availability

The data presented in this study are available on request from the corresponding authors.

## Supplementary Materials

The following supporting information can be downloaded at: https://www.mdpi.com/article/10.3390/ma16103779/s1, Figure S1: Unit cells of various Fe-C structures used in this study: (a) B1, (b) B2, (c) B3 and (d) L12. Fe atoms are denoted by large (blue) spheres whereas C atoms are denoted by smaller (black) spheres; Figure S2: Unit cells of various Fe-C structures used in this study: (a) Cementite, and (b) O-Fe${}_{7}$C${}_{3}$. Fe atoms are denoted by large (blue) spheres whereas C atoms are denoted by smaller (black) spheres; Table S1: Elastic Properties of B2, B3, and L12 structures calculated by the developed RF-MEAM potential.
