# Supplementary material for: Development of the RF-MEAM Interatomic Potential for the Fe-C System to Study the Temperature-Dependent Elastic Properties
Source: Materials (Basel). 2023 May 17;16(10):3779. doi: 10.3390/ma16103779 (PMC10222196; doi:10.3390/ma16103779)
Supplement: Supplementary file 1 [file materials-16-03779-s001.zip › materials-2393329-supplementary.pdf]

Supplementary Materials: Development of RF-MEAM Interatomic Potential for Fe-C System to Study Temperature Dependent Elastic Properties

Sandesh Risal [1], Navdeep Singh [2,*], Andrew Ian Duff [3], Yan Yao [4,5], Li Sun [1], Samprash Risal [4] and Weihang Zhu [1,6,*]

1. Structures of Fe-C Used in This Study

The structures of Fe-C used in this study are B1, B2, B3, L12, Cementite, and O-Fe_7C_3. B1, B2, B3, and L12 are all cubic structures commonly known as NaCl, CsCl, ZnS, and Cu_3Au structure respectively. Cementite and O-Fe_7C_3 are Orthorhombic structures. The unit cells for all these structures are given below in Figures S1 and S2

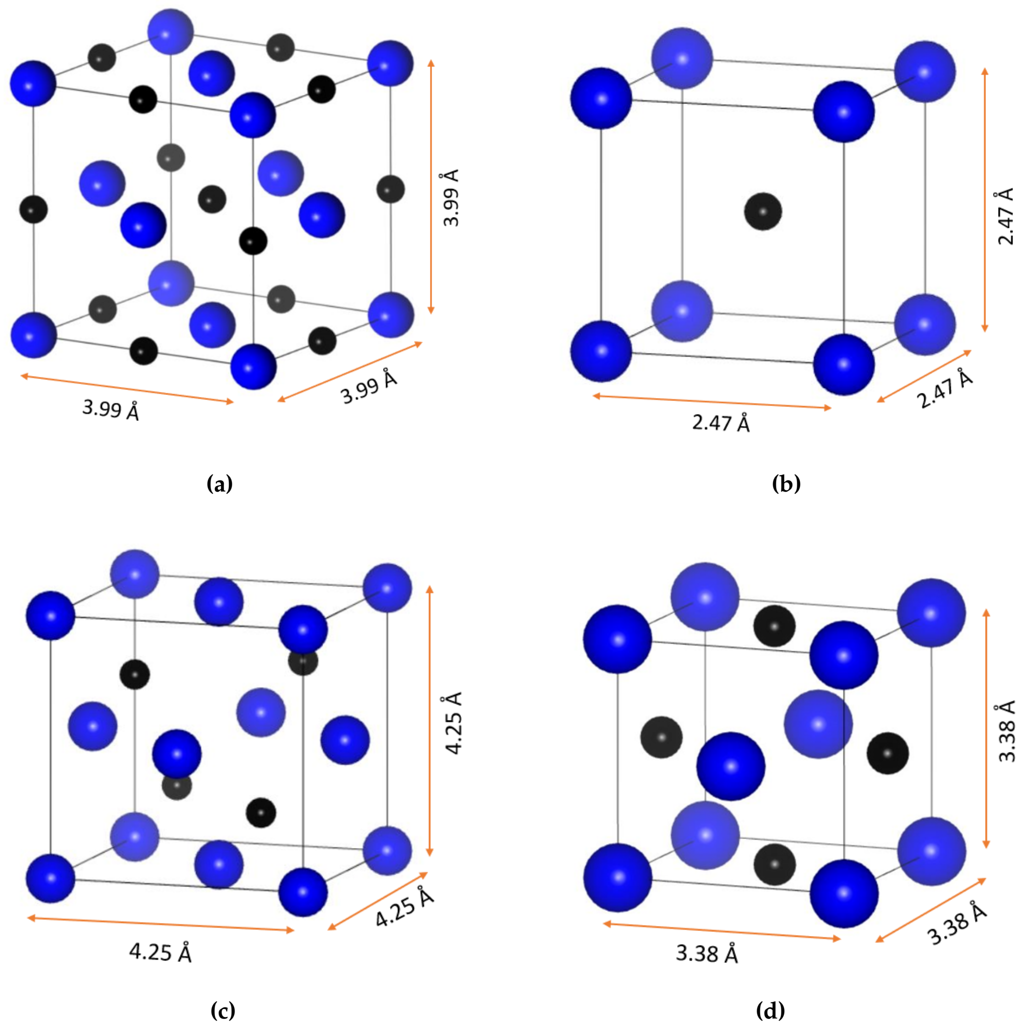

Figure S1. Unit cells of various Fe-C structures used in this study: (a) B1, (b) B2, (c) B3 and (d) L12. Fe atoms are denoted by large (blue) spheres whereas C atoms are denoted by smaller (black) spheres

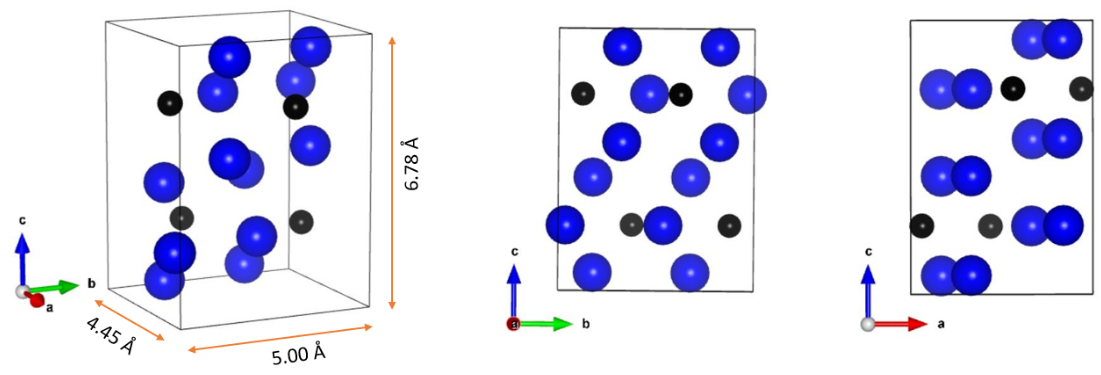

(a)

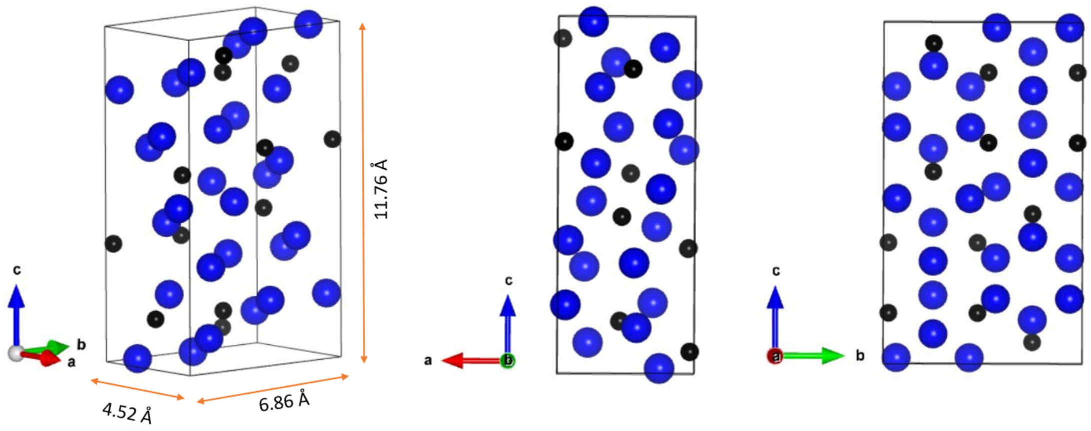

(b)

Figure S2. Unit cells of various Fe-C structures used in this study: (a) Cementite, and (b) O-Fe_7C_3. Fe atoms are denoted by large (blue) spheres whereas C atoms are denoted by smaller (black) spheres

2. Elastic Properties of B2, B3, and L12

The main text did not include information on the elastic properties of B2, B3, and L12 structures because there was no literature available for comparison. However, Table S1 displays the single crystal elastic constants (Cij) and the polycrystalline elastic constants for these three structures.

Table S1. Elastic Properties of B2, B3, and L12 structures calculated by the developed RF-MEAM potential.

Elastic Constants (GPa)	C11	C22	C33	C12	C13	C23	C44	C55	C66	B	G	Y
**B2**	540	655	583	258	239	286	133	99	141	372	141	375
**B3**	355	310	287	178	222	179	67	20	43	235	51	143
**L12**	309	309	309	188	188	188	89	89	89	228	77	209

3. Calculation of Polycrystalline Elastic Constants

The polycrystalline elastic constants, namely bulk modulus (B), rigidity modulus (G), and Young's modulus are calculated from stiffness tensors (C_{ij}), and compliance tensor (S_{ij}) using Voigt, Reuss, and Hill equations [1–3]. The compliance tensor S is calculated as the

inverse of the stiffness tensor, $[S_{ij}]$ = $[C_{ij}]^{-1}$. According to the Voigt [1] approximation, B, and G are expressed as:

$$B_V = \frac{1}{9}(c_{11} + c_{22} + c_{33}) + \frac{2}{9}(c_{12} + c_{13} + c_{23}) \tag{S1}$$

$$G_V = \frac{1}{15}(c_{11} + c_{22} + c_{33} - c_{12} - c_{13} - c_{23}) + \frac{1}{5}(c_{44} + c_{55} + c_{66}) \tag{S2}$$

According to the Reuss [2] approximation, B, and G are expressed as:

$$B_R = \frac{1}{(s_{11} + s_{22} + s_{33}) + 2(s_{12} + s_{13} + s_{23})} \tag{S3}$$

$$G_R = \frac{1}{4(s_{11} + s_{22} + s_{33}) - 4(s_{12} + s_{13} + s_{23}) + 3(s_{44} + s_{55} + s_{66})} \tag{S4}$$

The Voigt and Reuss approximation represent the upper and lower limit of the true polycrystalline modulus. Hill [3] proposed the arithmetic mean values of the Voigt's and Reuss' modulus expressed as:

$$B_H = \frac{1}{2}(B_R + B_H) \tag{S5}$$

$$G_H = \frac{1}{2}(G_R + G_H) \tag{S6}$$

The Young's modlus (Y) can be calculated using B_H, and $G_H$using the following equations.

$$Y_H = \frac{9B_H G_H}{3B_H + G_H} \tag{S7}$$

References

1. Voigt, W. Lehrbuch der Kristallphysik: Teubner-Leipzig. (Macmillan New York,1928)
2. Reuß, A. Berechnung der fließgrenze von mischkristallen auf grund der plastizitätsbedingung für einkristalle.. *ZAMM-Journal Of Applied Mathematics And Mechanics/Zeitschrift Für Angewandte Mathematik Und Mechanik*. **9**, 49-58 (1929)
3. Hill, R. The elastic behaviour of a crystalline aggregate. *Proceedings Of The Physical Society. Section A*. **65**, 349 (1952)
